# Comparing the Outcomes of Intramedullary Nail Fixation for Subtrochanteric Femur Fractures Performed by Trauma Fellowship-Trained and Non-Trauma-Trained Surgeons

**DOI:** 10.7759/cureus.114202

**Published:** 2026-08-09

**Authors:** Stephen M Himmelberg, Nathaniel T Koutlas, Abigail E Smith, Océane Mauffrey, Victoria J Garrard, Alysa B Nash, Daniel S Whittingslow, Andrew T Chen

**Affiliations:** 1 Orthopedic Surgery, University of North Carolina Hospitals, Chapel Hill, USA; 2 Orthopaedic Surgery, University of North Carolina Hospitals, Chapel Hill, USA; 3 Orthopaedics, University of North Carolina, Chapel Hill, USA

**Keywords:** proximal femur fracture, subtrochanteric, subtrochanteric femur fracture, trauma, trauma and orthopedic surgery

## Abstract

Introduction: Reduction of subtrochanteric femur fractures can be challenging due to the strong deforming forces at the hip. Increased experience with trauma fellowship training may potentially result in better outcomes, but subspecialty-trained surgeons may not be available for each case.

Purpose: The goal of this study was to compare postoperative complication rates, radiographic outcomes, and union rates among trauma fellowship-trained and non-trauma-trained orthopaedists following intramedullary nail fixation (IMN) of subtrochanteric femur fractures.

Methods: All patients with a subtrochanteric femur fracture treated at an academic Level 1 trauma center between 2010 and 2020 were identified. One hundred and thirty patients met the inclusion criteria and had a minimum follow-up of three months. Medical records were reviewed for demographic data, comorbidities, and associated injuries. Intraoperative variables, including implant, positioning, and the need for open reduction, were collected. Complications, including nonunion, surgical site infection (SSI), and reoperation, were compared between the two cohorts.

Results: Seventy-one patients were treated by trauma surgeons, while 59 patients were treated by non-trauma surgeons. Patients treated by trauma surgeons were more likely to be younger (58.1 vs 67.5, p = 0.01) and male (60.6% vs 44.1%, p = 0.06). Initial Injury Severity Score was similar (14.2 vs 13.1, p = 0.51). Open reduction was performed more frequently by non-trauma surgeons (18.3% vs 44.1%, p < 0.0014). The overall union rate was not statistically different (96% vs 90.3%, p = 0.36), while there was a trend toward malunion and malreduction in the non-trauma-trained cohort; these differences were not statistically significant (19% vs 6%, p=.092; 20% vs 10%, p=0.079). Reoperation rates (11.3% vs 8.5%, p=0.59), SSI (2.8% vs 5.1%, p=0.50), and readmission rates (2.8% vs 8.5%, p=0.15) were not statistically different.

Conclusion: Union rates were similar between surgeons with and without trauma fellowship training after intramedullary nailing of subtrochanteric femur fractures. Further prospective studies are required to remove any inherent bias related to injury complexity.

## Introduction

Subtrochanteric femoral fractures are becoming more common and exhibit a bimodal distribution [1]. The incidence has been estimated at 15-20/100,000 patients, accounting for 5-10% of all proximal femur fractures [1,2]. These fractures are at high risk of nonunion, with a rate of 4-5% despite significant technological advances and a shift away from plate fixation toward intramedullary implants [3,4]. Several fracture-specific factors contribute to the elevated nonunion risk, namely malreduction, primarily attributable to the strong muscular deforming forces at the fracture making it difficult to obtain and maintain an acceptable reduction[3,5]. Malreduction with >10 degrees of angulation in any plane or displacement on AP or lateral radiographs has been associated with higher rates of delayed union and nonunion, underscoring the importance of adequate reduction [6,7]. Malunions and nonunions are poorly tolerated in the subtrochanteric region of the body due to the substantial forces distributed around the hip joint, resulting in shortening of the limb with subsequent pain, difficulty ambulating, and disability [3].

Studies in other surgical fields have demonstrated mixed results regarding the importance of subspecialty training on the clinical outcomes of various surgical procedures [8-11]. Within the field of orthopaedic trauma, prior research has investigated intertrochanteric femur fractures as well as femoral shaft fractures to analyze whether subspecialty training has an impact on radiographic outcomes. While trauma fellowship-trained orthopaedic surgeons achieved better reductions of intertrochanteric hip fractures than their counterparts, a separate paper analyzing femoral shaft fractures found no differences in malrotation or femoral version between trauma surgeons and non-trauma fellowship-trained surgeons [12,13].

To date, no studies have analyzed the impact of subspecialty training on postoperative outcomes of subtrochanteric femoral fractures treated with an intramedullary nail, despite the propensity for malreduction. The primary outcome of this study was to compare the rates of union, malunion, and nonunion between orthopaedic surgeons with and without trauma fellowship training. Secondary outcomes of this study included comparisons of complication rates, surgical techniques, and fixation strategies between the two cohorts. Due to the known difficulties with malreduction and nonunion in subtrochanteric femur fractures, we hypothesized that trauma fellowship-trained orthopaedists would have lower rates of nonunion and malunion, along with a lower complication rate, following intramedullary nailing of subtrochanteric femur fractures compared to non-trauma fellowship-trained surgeons.

## Materials and methods

This was a retrospective study of all surgically treated cases of subtrochanteric femoral fractures in adults (≥18 years old) at an academic center from 2010 to 2020. Cases were identified by querying CPT code 27245 for operative treatment of proximal femur fractures with an intramedullary implant from our hospital's electronic medical record. The patients were grouped into one of two cohorts: those treated by a surgeon who had completed a one-year fellowship specifically in Orthopaedic Trauma, and those treated by a surgeon who had completed a one-year fellowship in a specialty other than Orthopaedic Trauma. Cases were then confirmed to be subtrochanteric femur fractures by retrospectively reviewing the injury radiographs. Subtrochanteric fractures were defined as proximal femur fractures occurring from the lesser trochanter to 5 cm distal into the proximal femoral shaft. Patients were required to have follow-up of at least three months, and exclusion criteria included pathologic fractures (oncologic and bisphosphonate-induced) and prior injury or deformity. Patient charts were reviewed to collect demographic data (age, sex, race, BMI, mechanism of injury) and pertinent medical comorbidities, as well as associated injuries. Operative reports and postoperative clinic notes were reviewed to record the attending surgeon's training, type of fixation used, fracture outcome, reoperation, and readmission related to surgery. Intraoperative and initial postoperative radiographs were reviewed for acceptable reduction (<10 degrees of malalignment in the coronal and sagittal planes). For fracture outcomes, union was defined as any bony union, including malunion, as evidenced by bridging bone or cortical continuity across a minimum of 3 of 4 cortices on AP and lateral radiographs. Nonunion was defined as the absence of the previously stated union criteria, along with a lack of progressive fracture healing after six months from initial fracture management or hardware failure requiring revision surgery. Malunion was defined as any malalignment in the coronal or sagittal plane greater than 10 degrees. Any patient without a minimum of six months of follow-up was not included in the fracture outcomes portion of the study. The Injury Severity Score was calculated for each case. Comparative analysis of our main and secondary outcome measures between trauma surgeons and non-trauma surgeons was performed using a chi-squared test, as well as the ANOVA F test, with a p-value of 0.05 used to determine significance.

## Results

Initial database queries identified 332 cases. Subsequent review excluded 202 cases on the basis of pathologic fracture, prior ipsilateral femur fracture, and failure to meet the minimum follow-up of three months. This yielded 130 total cases of subtrochanteric femur fractures treated operatively, with 71 treated by trauma orthopaedic surgeons and 59 treated by non-trauma-trained orthopaedic surgeons. Demographic data showed no significant differences in sex, BMI, race, or ethnicity (Table 1, p > 0.05). Mean age was statistically different between the two cohorts, with the trauma orthopaedists treating, on average, younger patients compared to their non-trauma-trained counterparts (Table 1; 58.1 vs 67.5, p < 0.05). 

**Table 1:** Demographic data ^1^Chi-square p-value. ^2^T-test p-value p<0.05 is considered statistically significant.

Variable	Trauma orthopaedists (N = 71)	Non-trauma orthopaedists (N = 59)	Overall (N = 130)	Statistic	Unadjusted p-value
Sex				χ² = 3.52	0.06061
Male	43 (60.6%)	26 (44.1%)	69 (53.1%)		
Female	28 (39.4%)	33 (55.9%)	61 (46.9%)		
Age (years)				χ² = 6.8187	0.01012
Mean (SD)	58.1 (22.05)	67.5 (18.53)	62.4 (20.99)		
Median (min, max)	53.0 (20.0, 103.0)	69.0 (18.0, 100.0)	65.5 (18.0, 103.0)		
BMI at time of injury				χ² = 0.2955	0.58772
N (missing)	67 (4)	55 (4)	122 (8)		
Mean (SD)	27.9 (7.54)	27.2 (7.05)	27.6 (7.31)		
Median (min, max)	27.0 (14.6, 51.7)	25.4 (15.3, 45.2)	26.4 (14.6, 51.7)		
Race				χ² = 1.1438	0.56441
White	47 (66.2%)	44 (74.6%)	91 (70.0%)		
African American	15 (21.1%)	10 (16.9%)	25 (19.2%)		
Other	9 (12.7%)	5 (8.5%)	14 (10.8%)		
Ethnicity				χ² = 1.0537	0.59051
Hispanic	5 (7.0%)	2 (3.4%)	7 (5.4%)		
Not Hispanic	64 (90.1%)	56 (94.9%)	120 (92.3%)		
Missing	2 (3.0%)	1 (1.7%)	3 (2.3%)		

When comparing patient comorbidities and Injury Severity Scores between the two patient cohorts, no significant differences were observed (Tables 2, 3; p > 0.05). Similar rates of chronic kidney disease (CKD), osteoporosis, and smoking status were demonstrated within the two populations. The greatest difference observed in comorbidities was in the prevalence of diabetes. Non-trauma-trained orthopaedists had an insignificantly higher prevalence of diabetes among their patients at 34% (n = 20) compared to 22% (n = 16) in the trauma-trained orthopaedist population (Table 2; p > 0.05). Injury Severity Score was similar between the two populations, with a mean score of 14.2 in the trauma orthopaedist cohort and 13.1 in the non-trauma orthopaedist cohort (Table 3; p > 0.05). However, the mechanism of injury was statistically different between the two cohorts, with trauma-trained surgeons treating subtrochanteric fractures resulting from high-energy mechanisms more frequently (Table 3; 54% (n = 38) vs 35% (n = 21), p < 0.05). 

**Table 2:** Comorbidities and substance use ^1^Chi-square p-value. p < 0.05 is considered statistically significant.

Variables	Trauma orthopaedists (N = 71)	Non-trauma orthopaedists (N = 59)	Overall (N = 130)	Statistic	Unadjusted p-value
Diabetes				χ² = 2.2649	0.1761^1^
Yes	16 (22.5%)	20 (34.5%)	36 (27.9%)		
No	55 (77.5%)	38 (65.5%)	93 (72.1%)		
Missing	0	1	1		
Chronic kidney disease				χ² = 0.2138	0.9236^1^
Yes	5 (7.0%)	3 (5.1%)	8 (6.2%)		
No	66 (93.0%)	56 (94.9%)	122 (93.8%)		
Osteoporosis				χ² = 1.0283	0.4413^1^
Yes	13 (18.3%)	7 (11.9%)	20 (15.4%)		
No	58 (81.7%)	52 (88.1%)	110 (84.6%)		
Smoking status				χ² = 1.1396	0.5656^1^
Current use	17 (23.9%)	15 (25.4%)	32 (24.6%)		
Former use	11 (15.5%)	13 (22.0%)	24 (18.5%)		
Never use	43 (60.6%)	31 (52.5%)	74 (56.9%)		

**Table 3:** Injury severity ^1^Chi-square p-value. ^2^ T-test p-value. *p < 0.05 is considered statistically significant.

Variables	Trauma orthopaedists (N = 71)	Non-trauma orthopaedists (N = 59)	Overall (N = 130)	Statistic	Unadjusted p-value
Mechanism of injury*				χ² = 4.5074	0.0337^1^
High	38 (54.3%)	21 (35.6%)	59 (45.7%)		
Low	32 (45.7%)	38 (64.4%)	70 (54.3%)		
Missing	1 (1.4%)	0 (0.0%)	1 (0.8%)		
Injury severity score				x^2^ = 0.4185	0.5188^2^
Mean (SD)	14.2 (9.58)	13.1 (8.76)	13.7 (9.20)		
Median (min, max)	9.0 (4.0, 57.0)	9.0 (9.0, 48.0)	9.0 (4.0, 57.0)		
Isolated injury				χ² = 3.4225	0.0961^1^
Yes	42 (59.2%)	44 (74.6%)	86 (66.2%)		
No	29 (40.8%)	15 (25.4%)	44 (33.8%)		
Initial ICU admission				χ² = 0.2765	0.5990^1^
Yes	59 (83.1%)	51 (86.4%)	110 (84.6%)		
No	12 (16.9%)	8 (13.6%)	20 (15.4%)		
Time to surgery (days)				x^2^ = 1.4992	0.2231^2^
Mean (SD)	2.8 (10.71)	1.1 (1.09)	2.0 (7.97)		
Median (min, max)	1.0 (0.0, 9.0)	1.0 (0.0, 7.0)	1.0 (0.0, 9.0)		

As shown in Table 4, non-trauma orthopaedists more frequently used a cephalomedullary nail (CMN) with a single-screw or two-screw construct, 65% (n = 37) of the time, compared to trauma-trained surgeons, who only used a CMN 38% (n = 26) of the time (Table 4, p < 0.05). Trauma surgeons preferred to use a reconstruction nail (RCN) with two proximal interlocking screws 62% (n = 42) of the time, compared to the non-trauma cohort, which used an RCN only 35% (n = 20) of the time (Table 4, p < 0.05). Open reduction was performed more than twice as often by non-trauma orthopaedic surgeons, at a rate of 44% (n = 26), compared to 18% (n = 13) for their counterparts (Table 4, p < 0.05). Trauma surgeons also utilized the lateral position (Table 4; 49.3% (n = 35) vs 3.4% (n = 2), p < 0.05) and a flat-top table (Table 4; 65.2% (n = 45) vs 1.8% (n = 1), p < 0.05) significantly more than the non-trauma-trained surgeons, who preferred supine positioning on a fracture table. 

**Table 4:** Surgical details ^1^Chi-square p-value. ^2^ANOVA F-test p-value. p<0.05 is considered statistically significant.

Variables	Trauma orthopaedists (N = 71)	Non-trauma orthopaedists (N = 59)	Overall (N = 130)	Statistic	Unadjusted p-value
Surgical time				F = 0.1060	0.1865^1^
N	71	59	130		
Mean (SD)	114.4 (77.72)	118.7 (69.86)	116.4 (74.00)		
Median (range)	95.0 (31.0, 502.0)	104.0 (41.0, 460.0)	97.5 (31.0, 502.0)		
Table used				χ² = 54.2360	<.0001^2^
Fracture	24 (34.8%)	56 (98.2%)	80 (63.5%)		
Flat top	45 (65.2%)	1 (1.8%)	46 (36.5%)		
Missing	2	1	3		
Positioning				χ² = 33.3508	<.0001^2^
Supine	36 (50.7%)	57 (96.6%)	93 (71.5%)		
Lateral	35 (49.3%)	2 (3.4%)	37 (28.5%)		
Need for open reduction				χ² = 10.1805	0.0014^2^
Yes	13 (18.3%)	26 (44.1%)	39 (30.0%)		
No	58 (81.7%)	33 (55.9%)	91 (70.0%)		
Implant used				χ² = 8.8274	0.0052^2^
Recon nail	42 (61.8%)	20 (35.1%)	62 (49.6%)		
Cephalomedullary nail	26 (38.2%)	37 (64.9%)	63 (50.4%)		
Number of distal interlocks				χ² = 0.1985	0.7903^1^
One	30 (42.9%)	23 (39.0%)	53 (41.1%)		
Two	40 (57.1%)	36 (61.0%)	76 (58.9%)		

Overall complication rates did not differ between the two cohorts, with both groups experiencing similar rates of surgical site infection, reoperation, and readmission (Table 5; p > 0.05). While trauma surgeons experienced a higher overall union rate (union and malunion), with 96% (n = 48) of patients achieving union at an average of 17 weeks, non-trauma orthopaedic surgeons achieved an overall 90% (n = 28) union rate at an average of 19 weeks, and these results were not statistically significant between the two cohorts (Table 6; p > 0.05). Results also demonstrated a notable but statistically insignificant higher rate of malreduction, at 20% (n = 12), in the non-trauma-trained orthopaedic cohort when compared to their trauma counterparts, who experienced malreduction in 10% (n = 7) of cases (Table 5, p > 0.05). This trend was also demonstrated when looking at malunion rates, which were higher at 19% (n = 6) in the non-trauma-trained group compared to 6% (n = 3) in the trauma-trained group, but again were not statistically significant (Table 6; p > 0.05). There were no significant differences in fracture morphology between the trauma-trained and non-trauma-trained cohorts (Table 7; p > 0.05). 

**Table 5:** Outcomes and complications ^1^Chi-square p-value. ^2^ANOVA F-test p-value. p<0.05 is considered statistically significant.

Variables	Trauma orthopaedists (N = 71)	Non-trauma orthopaedists (N = 59)	Overall (N = 130)	Statistic	Unadjusted p-value
Length of hospital stay (days)				x^2^ = 1.4198	0.2356^1^
Mean (SD)	10.4 (11.12)	8.4 (6.72)	9.5 (9.40)		
Median (Min, Max)	7.0 (1.0, 62.0)	6.0 (2.0, 35.0)	6.0 (1.0, 62.0)		
Mortality during admission				χ² = 2.4444	0.1179^2^
Yes	0 (0.0%)	2 (3.4%)	2 (1.5%)		
No	71 (100.0%)	57 (96.6%)	128 (98.5%)		
Time to union (weeks)				x^2^ = 1.2686	0.2642^1^
N (Missing)	45 (26)	22 (37)	67 (63)		
Mean (SD)	17.1 (7.18)	19.4 (8.66)	17.9 (7.70)		
Median (Min, Max)	14.0 (8.0, 36.0)	18.0 (8.0, 40.0)	16.0 (8.0, 40.0)		
Time to final follow up (months)				x^2^ = 2.4153	0.1226^1^
Mean	7.3	5.1	6.3		
Surgical site infection				χ² = 0.4481	0.5032^2^
Yes	2 (2.8%)	3 (5.1%)	5 (3.8%)		
No	69 (97.2%)	56 (94.9%)	125 (96.2%)		
Need for re-operation				χ² = 0.2793	0.5972^2^
Yes	8 (11.3%)	5 (8.5%)	13 (10.0%)		
No	63 (88.7%)	54 (91.5%)	117 (90.0%)		
Re-admission				χ² = 2.0245	0.1548^2^
Yes	2 (2.8%)	5 (8.5%)	7 (5.4%)		
No	69 (97.2%)	54 (91.5%)	123 (94.6%)		
Sagittal alignment (degrees)				x^2^ = 0.1252	0.7241^1^
Mean (SD)	4.1 (5.18)	4.4 (5.36)	4.3 (5.25)		
Median (Min, Max)	3.0 (0.0, 36.0)	3.0 (0.0, 25.0)	3.0 (0.0, 36.0)		
Coronal alignment (degrees)				x^2^ = 6.4506	0.0123^1^
Mean (SD)	3.2 (3.62)	5.0 (4.41)	4.0 (4.08)		
Median (Min, Max)	2.0 (0.0, 22.0)	4.0 (0.0, 19.0)	3.0 (0.0, 22.0)		
Malreduction				χ² = 2.8359	0.0922^2^
Yes	7 (9.9%)	12 (20.3%)	19 (14.6%)		
No	64 (90.1%)	47 (79.7%)	111 (85.4%)		
Malrotation				χ² = 0.8233	0.3642^2^
Yes	1 (1.4%)	0 (0.0%)	1 (0.8%)		
No	70 (98.6%)	58 (100.0%)	128 (99.2%)		
Missing	0	1	1		

**Table 6:** Fracture outcomes ^1^Chi-square p-value. p<0.05 is considered statistically significant.

Variables	Trauma orthopaedists (N = 50)	Non-trauma orthopaedists (N = 31)	Statistic	Unadjusted p-value
Union	0.96 (48/50)	0.90 (28/31)	OR = 2.57	0.366^1^
Malunion	0.06 (3/50)	0.19 (6/31)	OR = 0.27	0.079^1^
Nonunion	0.04 (2/50)	0.10 (3/31)	OR = 0.39	0.365^1^

**Table 7:** Fracture characteristics ^1^Chi-square p-value. p<0.05 is considered statistically significant. AO: Arbeitsgemeinschaft für Osteosynthesefragen (Association for the Study of Internal Fixation), OTA: Orthopaedic Trauma Association.

Variables	Trauma orthopaedists (N = 71)	Non-trauma orthopaedists (N = 59)	Overall (N = 130)	Statistic	Unadjusted p-value
AO/OTA classification				χ²(2) = 4.34	0.1140
A	36 (50.7%)	39 (66.1%)	75 (57.7%)		
B	25 (35.2%)	17 (28.8%)	42 (32.3%)		
C	10 (14.1%)	3 (5.1%)	13 (10.0%)		

## Discussion

To our knowledge, this is the first study investigating outcomes of trauma-trained and non-trauma-trained orthopaedic surgeons in the management of subtrochanteric femur fractures. Although it has been well established that higher-volume centers have better clinical outcomes than low-volume centers for morbidity and mortality related to proximal femur and hip fractures, surgeon subspecialty in the repair of subtrochanteric femur fractures has not been specifically analyzed [14]. It has been demonstrated that the optimal timing for treatment of proximal femur fractures is within 24 to 48 hours in order to decrease morbidity and mortality [15]. Studies have similarly emphasized the importance of minimizing time to surgery for other hip fracture patterns [16,17]. Subtrochanteric femur fractures are a relatively common fracture morphology, accounting for up to 10% of proximal femur fractures [1,2]. The prevalence of this fracture type, combined with the importance of timing of operative fixation, makes subtrochanteric femur fractures a reasonable and expected call case for the on-call orthopaedic surgeon. Hypothetically, trauma fellowship training may result in better outcomes, but trauma fellowship-trained surgeons may not always be readily available. The goal of our study was to compare whether trauma fellowship training impacts outcomes and complication rates following intramedullary nail fixation of subtrochanteric femur fractures.

The cohorts were largely similar in demographic makeup and Injury Severity Score, with a nonsignificant difference in average age. Trauma-trained surgeons had a younger overall population, but time to surgery did not differ between the two groups. While there was a significant difference in open reduction, with non-trauma-trained surgeons performing open reduction twice as frequently, this did not significantly affect outcomes. Union rates, time to union, and reoperation rates showed no statistically significant differences. Furthermore, no increase in complications or readmission rates was seen between the two cohorts.

Previous literature has compared trauma subspecialty training to that of surgeons without trauma subspecialty training in patients who sustained intertrochanteric hip fractures [18]. Results showed no difference in mortality or Modified Barthel Index score; the authors reported significant differences in duration of surgery and time to surgery between the groups, with no effect on postoperative complication rates [18]. The reduction quality in intertrochanteric hip fractures between trauma subspecialty-trained and non-trauma-trained surgeons was also analyzed. In a retrospective cohort study of 200 patients, Haidukewych et al. found significantly higher rates of reductions classified as good among the trauma surgeon population and a significantly higher rate of reductions classified as bad among community surgeons [13]. This suggests that while reduction quality differs between the populations, this may not impact mortality or complication rates. Our study yielded similar outcomes, with no increase in complications and similar union rates between the two cohorts, but a trend toward malunion and malreduction in the non-trauma orthopaedist cohort, with a significant difference in increased coronal angulation.

Similar studies within the orthopaedic literature have analyzed the impact of subspecialty training on cost and clinical outcomes. A large database study found that pediatric-trained surgeons had similar rates of postoperative adverse events following posterior spinal fusion for adolescent idiopathic scoliosis but had lower blood loss and rates of blood transfusion than non-pediatric-trained surgeons [19]. In the treatment of pediatric supracondylar humerus fractures, there do not appear to be significant differences in malreduction, loss of reduction, or complication rates between pediatric-trained and non-pediatric-trained surgeons [20]. In adult reconstruction literature, a multicenter retrospective review demonstrated that the 90-day cost of total hip arthroplasty for femoral neck fractures was lower with surgeons trained in arthroplasty than with those without subspecialty training in joint replacement surgery [21]. Additionally, a retrospective review of 355 patients compared the difference in femoral version in femoral shaft fractures treated with intramedullary nails between trauma-trained and non-trauma-trained surgeons. They found no significant differences between the two groups [12].

The present study demonstrated no obvious differences in union outcomes when comparing orthopaedic surgeons with and without trauma fellowship training. The two cohorts had similar nonunion, infection, reoperation, and other complication rates. Our data suggest that, while there was a trend toward malreduction and malunion among non-trauma-trained surgeons in the treatment of subtrochanteric femur fractures, this did not reach statistical significance, and there was no difference in complication rates; however, trends in radiographic outcomes warrant further study. These findings support that timely fixation of subtrochanteric femur fractures can reasonably be pursued by a non-trauma fellowship-trained orthopaedic surgeon without increases in nonunion, malunion, or complication rates, which potentially may limit patient morbidity and mortality [22].

While there was a statistically insignificant trend toward malunion and malreduction, this study is limited by a small patient population. Additionally, a longer follow-up period would likely provide a more accurate representation of true union and malunion values, as well as complication rates. Our study also may underrepresent practice variations between trauma-trained and non-trauma-trained orthopaedic surgeons on a national scale, as only data from a single Level 1 trauma center were collected. Furthermore, data were collected at only one Level 1 trauma center, which may not account for community practices. A potential bias exists regarding the complexity of fracture patterns. Some cases may have been preferentially assigned or delayed to trauma-trained surgeons based on fracture complexity, as represented by the delay to surgery and higher-energy mechanism of injury seen in our trauma-trained cohort, which would underestimate true differences in outcomes. Similarly, malunion and nonunion rates based on nail geometry and start point were not evaluated in this study, although these factors may affect malreduction rates. Other clinical outcomes, such as functional outcomes, were not accounted for, which is a limitation of this study.

## Conclusions

In conclusion, union rates were high and similar between subtrochanteric femur fractures treated by fellowship-trained trauma surgeons and those treated by the non-trauma-trained on-call orthopaedist. However, the potential for inherent bias, with more complex cases being preferentially assigned to trauma-trained surgeons, must be noted, and subsequent prospective or multicenter studies are warranted.
